# Design of Group IIA Secreted/Synovial Phospholipase A_2_ Inhibitors: An Oxadiazolone Derivative Suppresses Chondrocyte Prostaglandin E_2_ Secretion

**DOI:** 10.1371/journal.pone.0010914

**Published:** 2010-06-01

**Authors:** Jean-Edouard Ombetta, Natacha Thelier, Chang Zhi Dong, Stéphanie Plocki, Lydia Tsagris, François Rannou, France Massicot, Atimé Djimdé, Elissar El-Hayek, Yiming Shi, Françoise Heymans, Nohad Gresh, Caroline Chauvet

**Affiliations:** 1 Laboratoire de Chimie Organique, Faculté de Pharmacie, Université François Rabelais, Tours, France; 2 Laboratoire de Pharmacologie, Toxicologie et Signalisation Cellulaire, INSERM UMR-S-747, UFR Biomédicale des Saints Pères, Université Paris Descartes, Paris, France; 3 Equipe de Pharmacochimie, ITODYS, CNRS UMR7086, Université Paris Diderot, Paris, France; 4 Service de rééducation, AP-HP, Hôpital Cochin, Paris, France; 5 Laboratoire de Chimie-Toxicologie analytique et cellulaire, EA4463, Faculté de Pharmacie, Université Paris Descartes, Paris, France; 6 Laboratoire de Chimie et Biochimie Pharmacologique et Toxicologique, CNRS UMR8601, UFR Biomédicale des Saints Pères, Université Paris Descartes, Paris, France; Ohio State University, United States of America

## Abstract

Group IIA secreted/synovial phospholipase A_2_ (GIIAPLA_2_) is an enzyme involved in the synthesis of eicosanoids such as prostaglandin E_2_ (PGE_2_), the main eicosanoid contributing to pain and inflammation in rheumatic diseases. We designed, by molecular modeling, 7 novel analogs of 3-{4-[5(indol-1-yl)pentoxy]benzyl}-4*H*-1,2,4-oxadiazol-5-one, denoted **C1**, an inhibitor of the GIIAPLA_2_ enzyme. We report the results of molecular dynamics studies of the complexes between these derivatives and GIIAPLA_2_, along with their chemical synthesis and results from PLA_2_ inhibition tests. Modeling predicted some derivatives to display greater GIIAPLA_2_ affinities than did **C1**, and such predictions were confirmed by *in vitro* PLA_2_ enzymatic tests. Compound **C8**, endowed with the most favorable energy balance, was shown experimentally to be the strongest GIIAPLA_2_ inhibitor. Moreover, it displayed an anti-inflammatory activity on rabbit articular chondrocytes, as shown by its capacity to inhibit IL-1β-stimulated PGE_2_ secretion in these cells. Interestingly, it did not modify the COX-1 to COX-2 ratio. **C8** is therefore a potential candidate for anti-inflammatory therapy in joints.

## Introduction

Inflammation is a multi-faceted process involving numerous enzymes, such as phospholipases A_2_ (PLA_2_s) and cyclo-oxygenases (COXs) [1]. PLA_2_s catalyze the hydrolysis of cell-membrane glycerophospholipids at the *sn*-2 position leading to the generation of free fatty acids such as arachidonic acid. The later is subsequently metabolized into potent pro-inflammatory mediators such as eicosanoids (e.g. prostaglandin E_2_ [PGE_2_]) through a pathway involving COX-1 and COX-2 in part [2]. PGE_2_ is the main eicosanoid contributing to pain and inflammation in rheumatic diseases [3], [4]. Nonsteroidal anti-inflammatory drugs (NSAIDs) reduce the production of PGE_2_, which leads to a significant improvement in rheumatic symptoms. However, these drugs exhibit gastrointestinal toxicity mainly because of a marked decrease in COX-1 activity [4] and renal and blood pressure toxicities mainly because of a decrease in COX-2 activity. COX-1 is constitutively expressed in most tissues and appears to be responsible for maintaining normal physiological function. However, COX-2 is absent in most tissues under normal resting conditions but is induced in inflamed tissues and is responsible for increased PGE_2_ production. This activation has motivated the development of selective COX-2 inhibitors. However, these inhibitors also have severe side effects such as myocardial infarction [5], [6]. Overcoming this problem could involve the development of novel anti-inflammatory agents to efficiently inhibit the PLA_2_-dependent production of COX substrates without impairing the balance between COX-1 and COX-2.

PLA_2_s represent a growing family of enzymes of two main categories, intracellular and secreted. Among the 10 human secreted PLA_2_s (sPLA_2_s) known to date, the most studied is the non-pancreatic Group IIA, denoted GIIAPLA_2_, because of its involvement in the pathogenesis of many inflammatory diseases (for a review, see [7]). GIIAPLA_2_ was originally purified from the synovial fluid of patients with rheumatoid arthritis [8], [9], [10]. The number of rheumatoid arthritis-affected joints and the presence of destructive erosion correlate with the amount of GIIAPLA_2_ in the serum of patients [11]. Moreover, GIIAPLA_2_ induces an inflammatory response when injected in rabbit joints [12] and exacerbates rat adjuvant arthitis after intradermal injection [13].

The systemic implication of sPLA_2_s in inflammation has prompted a number of research groups to develop selective inhibitors of different types of these enzymes. Some potent candidates have been evaluated in phase II clinical trials. Surprisingly, no effect was observed when such inhibitors were used to treat patients with sepsis or rheumatoid arthritis [14], [15]. This failure could be due to the complexity of the inflammation process and the existence of compensatory pathways. However, these molecules have been tested only in high-level systemic inflammatory diseases, not in low-level inflammatory diseases such as atherosclerosis, diabetes, Alzheimer's, and osteoarthritis. Varespladib, a sPLA_2_ inhibitor, was recently found to reduce atherosclerosis in apolipoprotein-E-null mice [16]. Thus, the efficacy of sPLA_2_ inhibitors in these low-level inflammatory diseases should be re-examined.

We have developed various selective inhibitors of sPLA_2_s [17], [18], [19], [20], [21]. Previously, we reported on the computer-assisted design and synthesis of a series of novel oxadiazolone derivatives that were shown to exhibit potent inhibitory properties against GIIAPLA_2_ [20]. In this series, a Ca(II)-binding oxadiazolone ring was connected through a polymethylene chain of varying lengths to an indole ring, which has been shown to be involved in apolar and cation-π interactions with GIIAPLA_2_ residues. The optimal length of the linker was found to encompass 5 methylenes, and the corresponding compound, (3-{4-[5-(indol-1-yl)pentoxy]benzyl}-4*H*-1,2,4-oxadiazol-5-one), is denoted **C1** in the present study. In the current work, the indole moiety was replaced by other aromatic groups, which gave rise to compounds **C2** to **C8**. Using molecular modeling, we computed and ranked energy balances for the binding of these inhibitors to GIIAPLA_2_. The inhibitory potencies of **C2** to **C8** against GIIAPLA_2_ was analyzed by enzymatic assay, and the anti-inflammatory activity of the most potent compound, **C8**, was evaluated in IL-1β-treated articular chondrocytes.

## Results

### Molecular modeling

**Figure 1:**
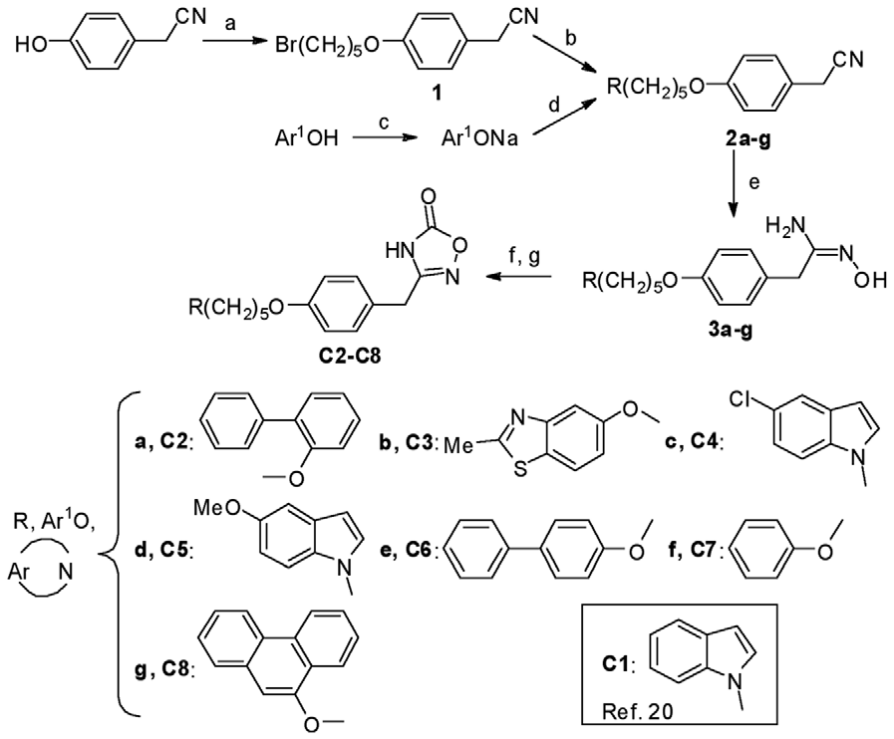
Synthesis scheme. Reagents and conditions: (a) Br(CH_2_)_5_Br, K_2_CO_3_, DMF, RT, 10 days; (b) Ar-NH, K_2_CO_3_, CH_3_CN, reflux; (c) NaOH, Abs EtOH; (d) 1, DMF, RT, 24 h; (e) NH_2_OH-HCl, K_2_CO_3_, Abs EtOH; (f) PhOCOCl, Et_3_N, CH_2_Cl_2_; (g) toluene, reflux. The terms **1**, **2a-g** and **3a-g** written in bold refer to the **C2-C8** precursors. The terms **a** to **g** written in bold in the bottom of the figure refer to the radicals (R) of the **C2** to **C8** compounds, respectively. The radical of the **C1** compound is also shown.

We previously reported that one of the essential interactions between **C1** and the target GIIAPLA_2_ is Ca(II) bidentate chelation by the oxadiazolone moiety in its anionic form [20]. Because **C2-C8** are structurally similar to **C1** (Fig. 1), docking was performed upon first anchoring the oxadiazolone ring in the same position as compound **C1**, followed by energy minimization and molecular dynamics. As was observed for compound **C1**, the lowest-energy frames of **C2-C8/**enzyme complexes are stabilized by π–π and cation-π interactions involving His6, Phe23, and Phe63 on the one hand and Arg7 and Arg33 of GIIAPLA_2_ on the other. Table 1 lists the energy values corresponding to the lowest-energy frames from molecular dynamics.

**Table 1:** Energy balances (ε = 4) from performing single-point Poisson-Boltzmann calculations of continuum solvation energies.

Cpd	E_int_	δE_prot_	δE_lig_	δE_1_	E_solv_	E_solvlig_	E_solvprot_	δE_solv_	δE_2_
**C1**	−129.5	5.5	8.1	−115.9	−344.3	−22.5	−385.1	63.3	−52.6
**C2**	−131.8	7.8	10.6	−113.4	−343.6	−23.9	−385.1	65.4	−48.0
**C3**	−129.6	6.0	8.7	−114.9	−344.1	−22.7	−385.1	63.7	−51.2
**C4**	−130.1	5.6	8.0	−116.5	−343.7	−21.7	−385.1	63.1	−53.2
**C5**	−131.2	5.1	9.9	−116.2	−350.0	−21.8	−385.1	56.9	−59.3
**C6**	−130.8	10.0	9.5	−111.3	−350.1	−21.7	−385.1	56.7	−54.6
**C7**	−122.6	5.1	8.5	−109.0	−340.2	−21.8	−385.1	66.7	−42.3
**C8**	−135.1	7.1	9.8	−118.2	−349.7	−21.8	−385.1	57.2	−61.0

All energies are given in kcal/mol. E_int_ denotes the inhibitor (compounds **C1** to **C8**)-protein interaction energy, and δE_lig_ and δE_prot_ the costs of conformational energy rearrangements of the inhibitor and the protein, respectively, on passing from their free to complexed states, and δE_1_ is the sum: E_int_+ δE_lig_ + δE_prot_. δE_1_ corresponds to a gas-phase complexation energy. E_solvprot_ and E_solvlig_ denote the continuum solvation energies of the isolated protein and the ligand, respectively, following gas-phase energy minimization in the absence of complexation. They represent the energy cost necessary to dehydrate both entities prior to complex formation. E_solvtot_ denotes the continuum solvation energy of the complex. Thus, δE_solv_  =  E_solvtot_ – E_solvlig_ – E_solvprot_, which represents the resulting solvation energy balance. The overall energy balance including solvation is denoted as δE_2_  =  δE_1_ + δE_solv_.

### Chemistry

As outlined in Figure 1, 4-(5-bromopent-1-yloxy)benzyl cyanide **1** is prepared according to Dehaen and Hassner [22] by mono-substitution of 1,5-dibromopentane with 4-hydroxybenzyl cyanide in moderate yield. Compound **1** is then condensed in 25% to 50% yields, with 5-substituted indole derivatives or different aromatic alcohols, through their sodium salts prepared prior to use, to give **2a–g**. The nitrile function of **2a–g** is converted into amidoxime, by use of hydroxylamine released *in situ* from its HCl salt, to provide **3a–g** in 35% to 80% yields. The action of phenyl chloroformate to the amidoximes **3a–g** leads to the corresponding carbonate intermediates, which, when heated to reflux of toluene, cyclizes intra-molecularly to generate the substituted oxadiazolones **C2-C8** in 34% to 50% yields.

### *In vitro* inhibition of enzymatic activity of sPLA_2_s by C1-C8

The compounds **C1-C8** were submitted to fluorimetric assay to determine their inhibitory potencies and selectivity towards human GIIAPLA_2_ (hGIIAPLA_2_) versus porcine group IB PLA_2_ (pGIBPLA_2_) (Table 2). GIBPLA_2_ is an enzyme of the same family as GIIAPLA_2_ (sPLA_2_) but is mainly involved in digestion of dietary phospholipids and is secreted by the pancreas [23]. Lipophilicity parameters, log P, of these products are calculated by use of Rekker's fragmental data [24] (Table 2). The molecules **C1-C8** are specific inhibitors of hGIIAPLA_2_ because none inhibited pGIBPLA_2_ at the highest concentration tested (100 µM). Such selectivity implies that **C1-C8** should not interfere with the digestion process.

**Table 2:** Inhibition of enzymatic activities of porcine pancreatic group IB (pGIB) and human group IIA (hGIIA) PLA_2_s by compounds **C1** to **C8** and their corresponding log *P* values.

Cpd	Log *P* *	IC_50_ (µM)	
		hGIIAPLA_2_	pGIBPLA_2_
**C1**	3.81	5.0±0.7	>100
**C2**	5.59	10.0±1.5	>100
**C3**	2.88	5.0±1.2	>100
**C4**	4.57	6.5±1.8	>100
**C5**	3.91	2.5±0.5	>100
**C6**	5.59	3.0±0.2	>100
**C7**	3.64	35±1.8	>100
**C8**	7.13	0.62±0.15	>100

*: calculated using the Rekker's hydrophobic fragmental constants

**Figure 2:**
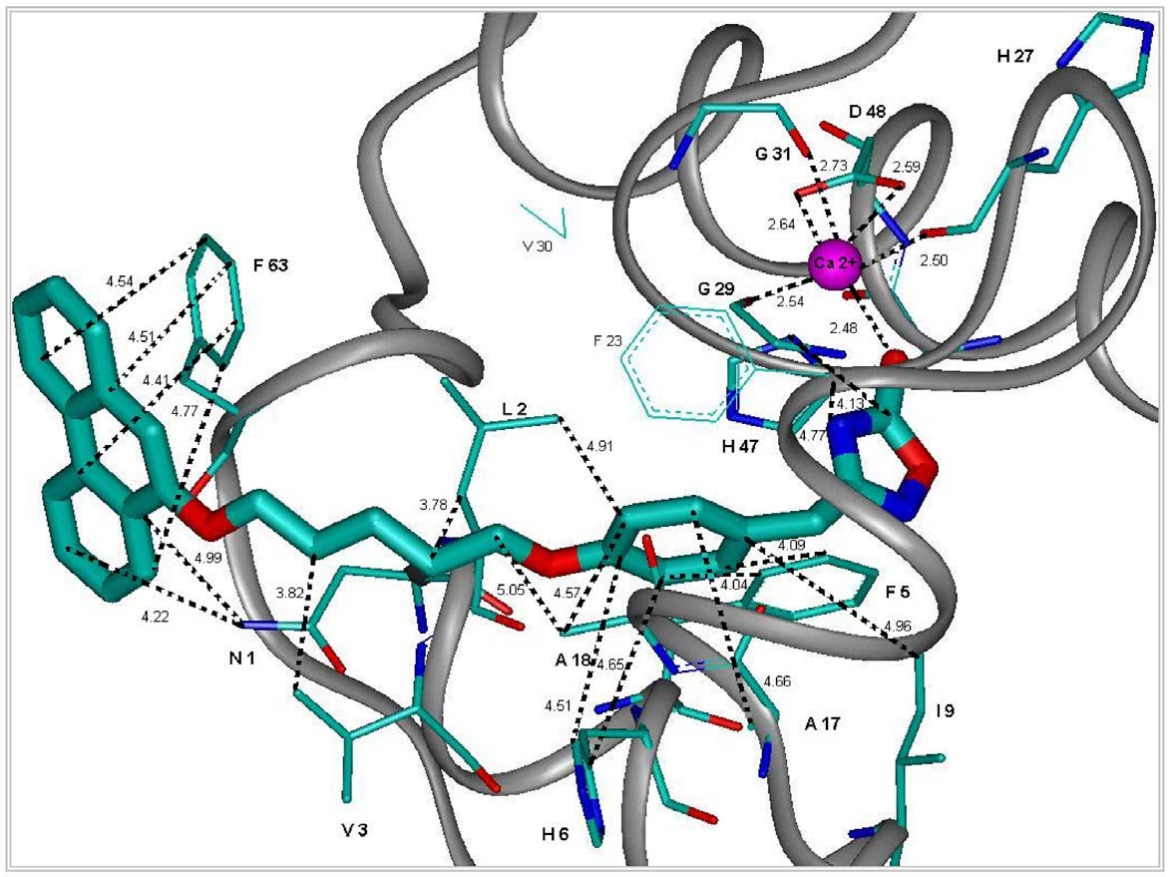
Representation of the most important interactions between C8 and the binding site of hGIIAPLA_2_ found from modeling. The structure presented in the figure was derived from molecular dynamics using the Accelrys software and the cff91 force field (see Supporting Information S1). In this presentation are shown in particular: a) the chelating of Ca(II) by the oxadiazolone moiety of **C8**, as well as Gly29 (G29), Gly31 (G31) and Asp48 (D48) of hGIIAPLA_2_ with their main-chain or side-chain carbonyls; b) the phenoxy binding site [(Leu2 (L2), Phe5 (F5), His6 (H6) and Ala18 (A18)]; and c) the binding site of 5-(phenanthren-9-yloxy)pentyl [Asn1 (N1), Val3 (V3) and Phe63 (F63)].

The experimentally measured IC_50_s for hGIIAPLA_2_ (Table 2) are associated with the final energy balances, denoted δE_2_ in Table 1. The ranking of **C1-C8** in terms of IC_50_ is the same as that of the δE_2_ magnitudes. In **C2**, the second phenyl ring is substituted with the ether O in the ortho position and in **C6** in the para position. Both IC_50_ and δE_2_ values show **C2** to have a significantly enhanced affinity for PLA_2_ as compared with **C6**, even though both are iso-lipophilic (Tables 1 and 2). In **C2**, the biphenyl group has favorable van der Waals interactions with both Phe23 and Val30 of the enzyme, but in **C6**, the interactions are limited to Phe23. Such interactions could be further optimized, as when the biphenyl ring was replaced by phenantrene in **C8**. The lowest-energy complex is now stabilized by an enhanced overlap of this ring with Phe63 (Fig. 2). However, the lipophilicity increases in parallel, which could possibly limit the bioavailability of **C8**. We found **C8** indeed endowed with the most favorable δE_2_ value (Table 1), which was experimentally associated with the lowest IC_50_ value (0.62 µM vs. 5 µM for **C1**).

At the other extreme, replacing the **C1** indole ring by the smaller and less electron-rich phenyl ring, as in **C7**, resulted in a reduction of 10.3 kcal/mol in δE_2_ value. Thus, **C7** can be predicted to have the least inhibitory potency in the series. This finding was confirmed by experimentation showing **C7** to have the highest IC_50_ value (35 µM vs. 5 µM for **C1**).

Similar to **C1**, compounds **C3-C5** have a bicyclic ring, whereas **C3** possesses a benzo-1,3-thiazole instead of an indole ring. **C4** and **C5** have a chlorine and a methoxy substituent, respectively, in position 5 of the indole. In **C3-C5**, the aromatic rings interact simultaneously with His6, Arg7, and Val3, as was previously observed for **C1** [20]. The difference in activity between **C4** and **C5** could be explained by additional electrostatic and/or van der Waals interactions contributed by methoxy substitution. **C3** has anti-hGIIAPLA_2_ activity close to that of **C1**, along with substantially reduced lipophilicity (2.88 vs. 3.81 for **C1**).

Thus, in the **C1**-**C8** series, **C8** has the most favorable δE_2_ value and the lowest IC_50_ on human GIIAPLA_2_ activity, as evaluated by enzymatic assay. On the bases of the IC_50_ values we focused our cellular assays on the most potent compound **C8**, the sole compound with a sub-micromolar activity. We thus chose to evaluate the cytotoxicity and anti-inflammatory activity of **C8** in primary cultured rabbit articular chondrocytes treated with the pro-inflammatory cytokine IL-1β, which is known to play a key role in rheumatic diseases such as osteoarthritis (for reviews see [25], [26]). Chondrocyte is the unique cell type in joints, and the cell model we chose is widely used to study the effect of inflammatory stress on joint cells.

### Evaluation of the cytotoxicity of C8 on articular chondrocytes

**Figure 3:**
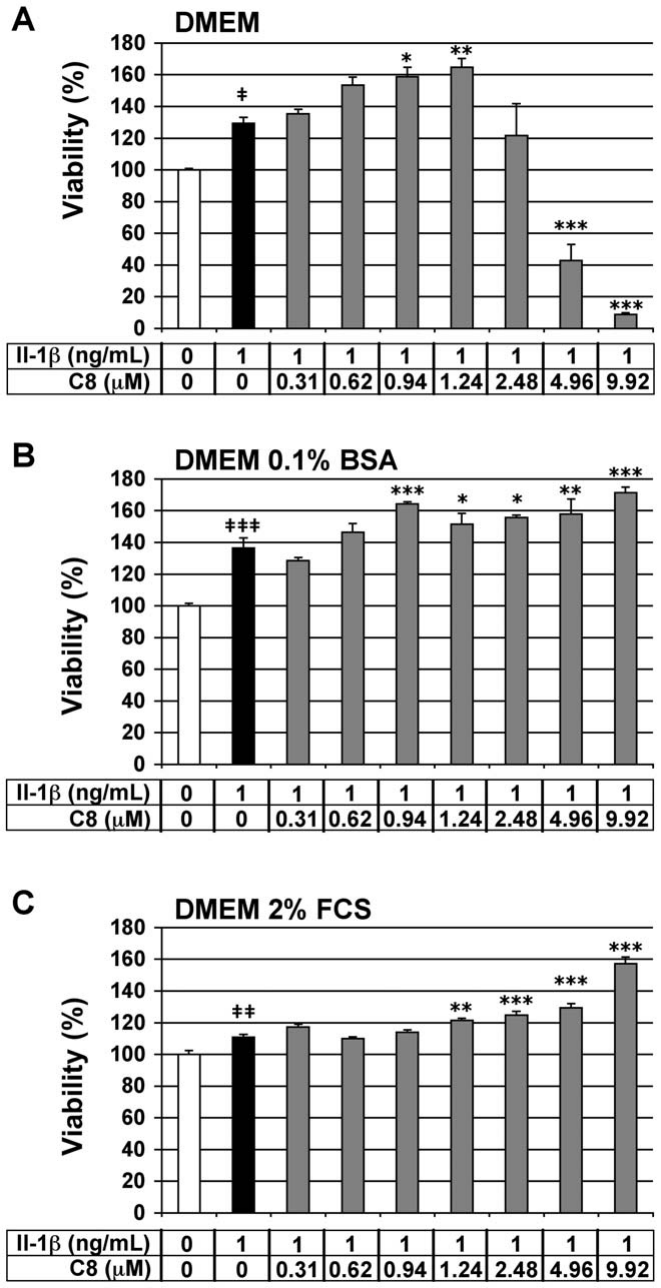
Effect of IL-1β and C8 on viability of articular chondrocytes. Chondrocytes were untreated (white bars) or treated for 20 h with IL-1β alone (black bars) or 1 h after the addition of **C8** (grey bars) in DMEM alone (A) or supplemented with 0.1% BSA (B) or 2% FCS (C). Cell viability was evaluated by an MTT-based test. Data represent the absorbance_570nm_ - absorbance_690nm_ and are expressed as relative arbitrary units, where the IL-1β-untreated group represents 100%. Values are means ± SEM (n = 3 to 8 independent determinations). **^‡^** *P*<0.05, **^‡‡^** *P*<0.01, **^‡‡‡^** *P*<0.001 between untreated and IL-1β-treated groups; * *P*<0.05, ** *P*<0.01, *** *P*<0.001 between IL-1β- and IL-1β+**C8**-treated groups.

We assessed the viability of the chondrocytes by MTT assay to evaluate the cytotoxic effects of **C8** on these cells. Chondrocytes were treated for 20 h with 1 ng/mL IL-1β alone or 1 h after the addition of **C8** at 0.31 to 9.92 µM, which corresponded to 0.5- to 5-fold the IC_50_ of **C8** on human GIIAPLA_2_ activity (Table 2). Three different culture medium compositions were used: DMEM alone, or supplemented with 0.1% BSA or 2% FCS. IL-1β had no cytotoxic effects as compared with the untreated control condition for the three culture media tested (Fig. 3). In chondrocytes cultured in DMEM alone but with IL-1β, **C8** had no cytotoxic effects at 0.31 to 2.48 µM (Fig. 3A). In chondrocytes cultured in DMEM with 0.1% BSA or 2% FCS and IL-1β, **C8** had no cytotoxic effects at 0.31 to 9.92 µM (Fig. 3B and 3C). Thus, we evaluated the anti-inflammatory activity of **C8** in culture conditions from 0.31 to 1.24 µM in DMEM alone and from 0.31 to 4.96 µM in DMEM supplemented with 0.1% BSA or 2% FCS.

### Effect of C8 on IL-1β-stimulated PGE_2_ secretion in articular chondrocytes

**Figure 4:**
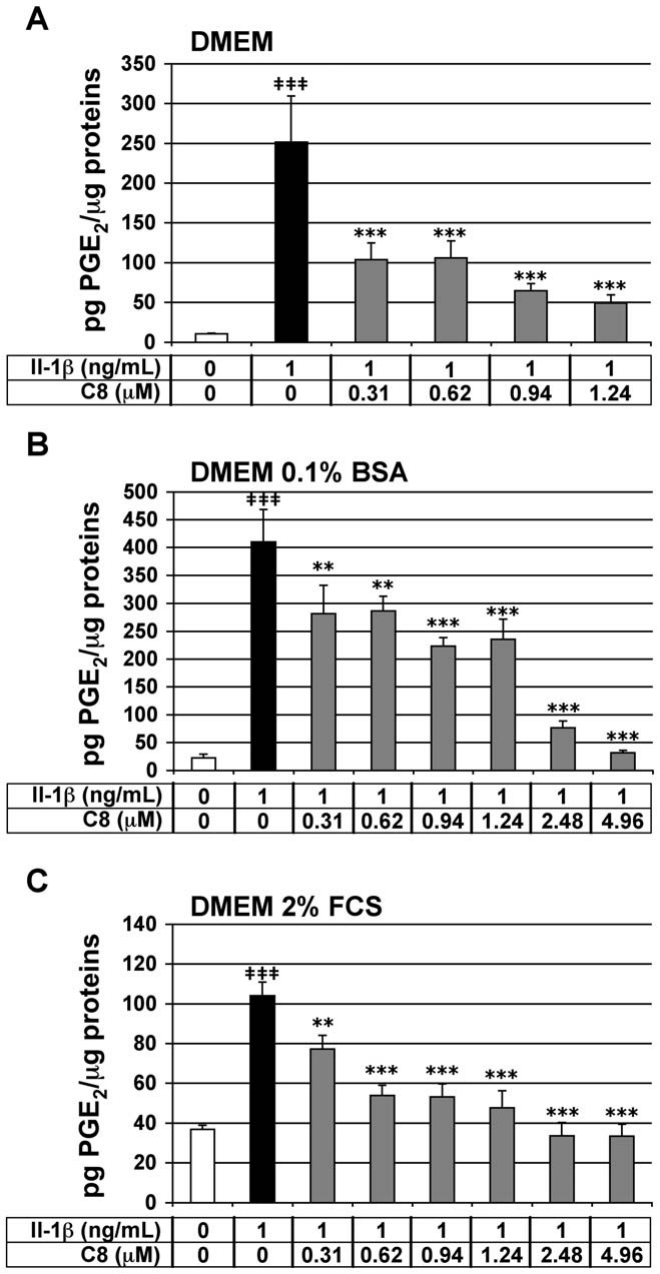
Effect of IL-1β and C8 on PGE_2_ secretion by articular chondrocytes. Chondrocytes were untreated (white bars) or treated for 20 h with IL-1β alone (black bars) or 1 h after the addition of **C8** (grey bars) in DMEM alone (A) or with 0.1% BSA (B) or with 2% FCS (C). PGE_2_ concentration was determined in conditioned culture medium, and protein concentration was determined in whole-cell protein extracts. Data represent the ratio of PGE_2_ concentration relative to whole cell protein concentration (pg PGE_2_/µg proteins). Values are means ± SEM (n = 3 to 7 independent determinations). **^‡^** *P*<0.05, **^‡‡^** *P*<0.01, **^‡‡‡^** *P*<0.001 between untreated and IL-1β-treated groups; * *P*<0.05, ** *P*<0.01, *** *P*<0.001 between IL-1β- and IL-1β+**C8**-treated groups.

We tested the effect of **C8** on the IL-1β-stimulated secretion of PGE_2_ in chondrocytes. PGE_2_ synthesis takes place mainly in response to cell activation by IL-1β, and its generation accounts for many of the actions induced by this cytokine [27]. *In vitro*, IL-1β induces the expression of COX-2 by chondrocytes, which results in increased PGE_2_ production [28]. PGE_2_ release thus represents a powerful IL-1β- and PLA_2_-dependent inflammatory marker in our cell model. Chondrocytes were treated for 20 h with IL-1β alone or 1 h after the addition of **C8**. As expected, IL-1β significantly stimulated PGE_2_ secretion by chondrocytes in the three different culture media: 23.3-, 18.3- and 2.8-fold induction as compared with untreated control conditions, in DMEM alone or supplemented with 0.1% BSA or 2% FCS, respectively (Fig. 4). In chondrocytes treated with IL-1β, **C8** had a strong and statistically significant inhibitory effect on PGE_2_ secretion at all concentrations tested: from 0.31 to 1.24 µM in DMEM alone or from 0.31 to 4.96 µM in DMEM supplemented with 0.1% BSA or 2% FCS (Fig. 4). In DMEM alone, at concentrations of 0.31-, 0.62-, 0.94-, and 1.24-µM, **C8** decreased the production of PGE_2_ induced by IL-1β by 59-, 58-, 74-, and 80-%, respectively (Fig. 4A). In DMEM supplemented with 0.1% BSA, at concentrations of 0.31-, 0.62-, 0.94-, 1.24-, 2.48-, and 4.96-µM, **C8** decreased the production of PGE_2_ induced by IL-1β by 31-, 30-, 45-, 43-, 81-, and 92-%, respectively (Fig. 4B). In DMEM supplemented with 2% FCS, at concentrations of 0.31-, 0.62-, 0.94-, 1.24-, 2.48-, and 4.96-µM, **C8** decreased the production of PGE_2_ induced by IL-1β by 26-, 48-, 49-, 54-, 68-, and 68-%, respectively (Fig. 4C). It is important to note that **C8** down-regulated the IL-1β-stimulated secretion of PGE_2_ to the level of the control untreated condition at 4.96 µM in DMEM supplemented with 0.1% BSA and at 2.48 and 4.96 µM in DMEM supplemented with 2% FCS. The effect of **C8** was then evaluated at the extreme concentrations (0.31- and 4.96-µM) in DMEM supplemented with 2% FCS and containing decreasing (1-, 0.5-, and 0.25-ng/mL) IL-1β concentrations (Table 3). The anti-IL-1β inhibitory effect of **C8** at 0.31 µM increases when IL-1β concentration decreases. The inhibitory effect of **C8** at 4.96 µM does not change when IL-1β concentration decreases. This is probably due to the fact that at 4.96 µM, the inhibitory effect of **C8** on IL-1β-induced PGE_2_ production is maximal. A parallel cellular test was performed on the compound **C1** whose IC_50_ is 5 µM (Table 2) and we observed that a 8 µM dose of **C1**, corresponding to 1.6-fold the IC_50_ of **C1** on human GIIAPLA_2_ activity, does not decrease the stimulated PGE_2_ secretion by IL-1β at 1 ng/mL (data not shown). Thus, **C8**, but not **C1**, decreases the IL-1β-stimulated PGE_2_ secretion in a dose-dependent manner in the three culture medium compositions used.

**Table 3:** Effect of **C8** on PGE_2_ secretion by articular chondrocytes incubated with different IL-1β concentrations.

	C8 inhibitory effect (%inhibition)	
IL-1β (ng/mL)	C8 at 0.31 µM	C8 at 4.96 µM
**1**	25*	68***
**0.5**	60*	67*
**0.25**	67	65

Chondrocytes were untreated or treated for 20 h with IL-1β (1-, 0.5-, or 0.25-ng/mL) alone or 1 h after the addition of **C8** (0.31- or 4.96-µM) in DMEM with 2% FCS. PGE_2_ concentration was determined in conditioned culture medium, and protein concentration was determined in whole-cell protein extracts. The PGE_2_ concentration was normalized relatively to whole cell protein concentration (pg PGE_2_/µg proteins). The means of PGE_2_ concentrations from 3 independent determinations were calculated and the anti-IL-1β inhibitory effect of **C8** was determined with the formula %inhibition  = 100−100×[(mean PGE_2_ in IL-1β+**C8** condition)/(mean PGE_2_ in IL-1β condition)]).

*P<0.05,

***P<0.001 between IL-1β- and IL-1β+**C8**-treated groups.

### Effect of C8 on IL-1β-stimulated NO secretion in articular chondrocytes

**Figure 5:**
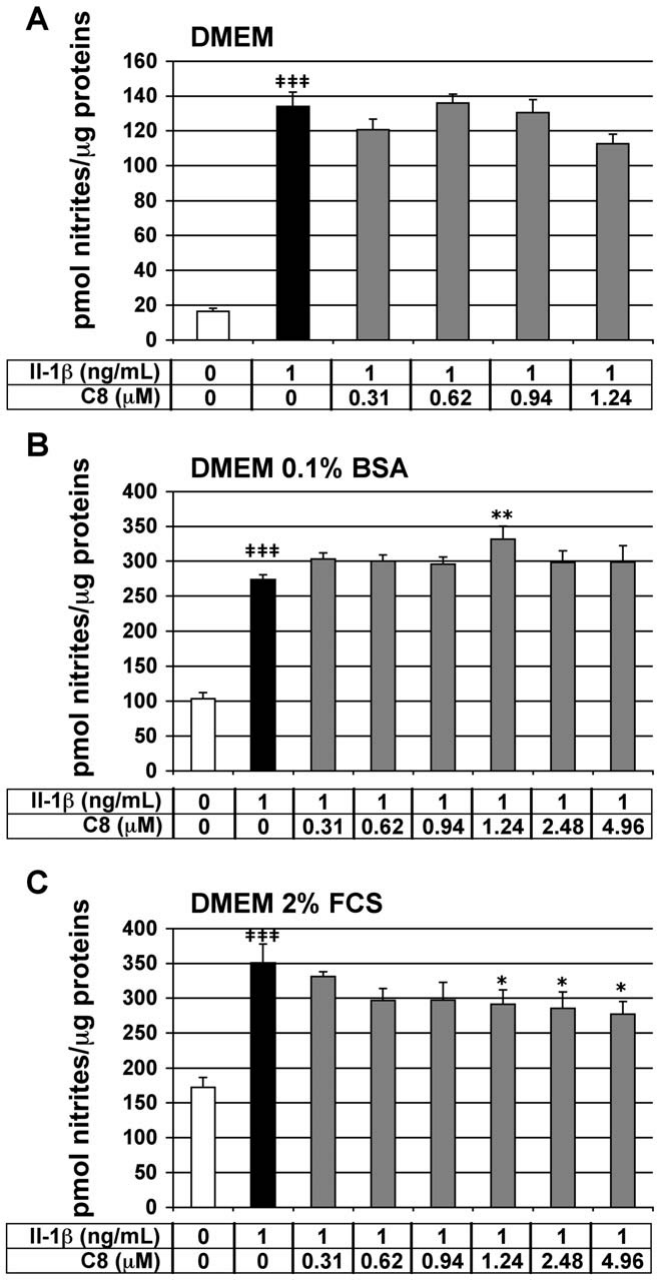
Effect of IL-1β and C8 on nitrite secretion by articular chondrocytes. Chondrocytes were untreated (white bars) or treated for 20 h with IL-1β alone (black bars) or 1 h after the addition of **C8** (grey bars) in DMEM alone (A) or supplemented with 0.1% BSA (B) or 2% FCS (C). NO secretion was indirectly evaluated by determination of nitrite concentration in conditioned culture medium. Protein concentration was determined in whole-cell protein extracts. Data represent the ratio of nitrite concentrations relative to whole-cell protein concentration (pmol nitrites/µg protein). Values are means ± SEM (n = 4 to 7 independent determinations). **^‡^** *P*<0.05, **^‡‡^** *P*<0.01, **^‡‡‡^** *P*<0.001 between untreated and IL-1β-treated groups; * *P*<0.05, ** *P*<0.01, *** *P*<0.001 between IL-1β- and IL-1β+**C8**-treated groups.

We tested the effect of **C8** on the IL-1β-stimulated secretion of NO in chondrocytes. NO is a mediator of immune and inflammatory responses. *In vitro*, IL-1β induces the expression of inducible NO synthase (iNOS) by chondrocytes, and consequently an increase in NO production [29]. NO secretion, evaluated by nitrite concentration in the cell culture medium, represents a reliable IL-1β-dependent and PLA_2_-independent inflammatory marker in our cell model. Chondrocytes were treated for 20 h with IL-1β alone or 1 h after the addition of **C8**. As expected, IL-1β significantly stimulated nitrite secretion by chondrocytes in the three different culture media: 8.1-, 2.6-, and 2.0-fold induction as compared with the control conditions, in DMEM medium alone or supplemented with 0.1% BSA or 2% FCS, respectively (Fig. 5). **C8** did not significantly inhibit the IL-1β-stimulated nitrite secretion in chondrocytes cultured in DMEM medium alone or supplemented with 0.1% BSA (Fig. 5A, B). In DMEM supplemented with 2% FCS, **C8** did not inhibit the IL-1β-stimulated nitrite secretion at 0.31-, 0.62-, and 0.94-µM and slightly decreased by 17-, 19-, 21-% the IL-1β-induced nitrite production at 1.24-, 2.48-, and 4.96-µM, respectively (Fig. 5C). Thus, **C8** did not inhibit the IL-1β-stimulated NO secretion in DMEM alone or supplemented with 0.1% BSA and slightly inhibited IL-1β-stimulated NO secretion in DMEM supplemented with 2% FCS.

### Effect of C8 on COX-1, COX-2, and iNOS protein levels in articular chondrocytes

**Figure 6:**
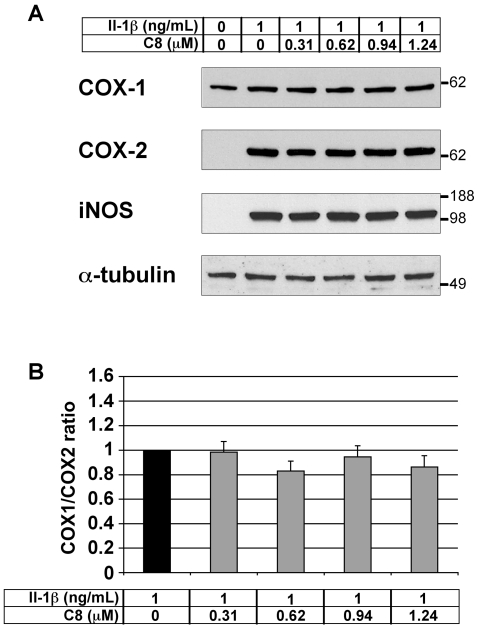
Effect of IL-1β and C8 on the COX-1, COX-2, and iNOS protein levels in articular chondrocytes. (A) Chondrocytes were untreated or treated for 20 h with IL-1β alone or 1 h after the addition of **C8** in DMEM. 20 µg aliquots of whole-cell protein extracts were examined by western blot analysis with antibodies against COX-1, COX-2, and iNOS. α-tubulin immunodetection is shown as a control for protein loading and transfer. Results from one representative experiment in five are shown. (B) Intensities of the COX-1 and COX-2 immunoreactive bands evaluated by semi-quantitative scanning densitometry. Data represent the COX-1/COX-2 protein ratio and are expressed as relative arbitrary units, where the IL-1β-treated group represents 1. Values are means ± SEM (n = 5 independent determinations). No significant differences were found between the groups.

We evaluated the effect of **C8** on COX-1, COX-2 and iNOS protein levels in chondrocytes treated with IL-1β. Chondrocytes were treated for 20 h with IL-1β alone or 1 h after the addition of **C8** (0.31-1.24 µM) in DMEM, and protein extracts were examined by western blot analysis. As expected, COX-1 protein was detectable but COX-2 and iNOS proteins were undetectable in untreated control conditions (Fig. 6A). Moreover, IL-1β treatment induced the expression of COX-2 and iNOS proteins without affecting the level of COX-1 protein (Fig. 6A). In the presence of IL-1β, **C8** did not alter the COX-1, COX-2 and iNOS protein levels (Fig. 6A). Consequently, the protein ratio of COX-1 to COX-2 was not modified by **C8** (Fig. 6B).

## Discussion

Nonsteroidal anti-inflammatory drugs (NSAIDs), which inhibit COX-1 and COX-2, and selective COX-2 inhibitors are currently used to reduce rheumatic symptoms. However, these drugs exhibit gastrointestinal, renal, blood pressure and cardiovascular toxicities. To overcome this problem, GIIAPLA_2_ inhibitors could be developed to inhibit the production of COX substrates without impairing the balance between COX-1 and COX-2. We designed and synthesized 7 new oxadiazolone derivatives (**C2** to **C8**) derived from **C1**. Using molecular modeling, we computed and ranked energy balances for the binding of these inhibitors to GIIAPLA_2_. The energy balances (Table 1) taking into account solvation effects show a correlation between δE_2_, the overall energy balance for binding, and the experimentally measured IC_50_ for our novel compounds **C1-C8**. This finding should lend additional credence to our previous results [20], despite the approximations of the computational approach used in that study, which allows for only single-point computations of Poisson-Boltzmann solvation energies for the most stable minima of the molecular dynamics procedure. Our useful predictions made with the present simplified energy potential may be due to the very local changes we made in the **C1-C8** series. These bear on the series' sole terminal aromatic group and target a limited number of amino acids, so that the accuracy of the energy potential may be sufficient. We plan to study such energy balances with the polarizable molecular mechanics procedure SIBFA [30], which, along with the Langlet-Claverie methodology for Continuum solvation [31], was recently used to investigate the binding of inhibitors to metalloenzymes [32]. This study should also allow for considering changes on other parts of the drugs as well.

One possible unfavorable feature of **C8** is its enhanced lipophilicity as compared with the other compounds. Nevertheless, this feature did not prevent the pharmacological efficiency of **C8** in chondrocytes. Reduction in Log P could be anticipated by replacing phenantrene with heterocyclic analogs and/or substitution with hydrophilic groups. Such reductions were seen on passing from compound **C1** with an indole ring to **C3** with a benzothiazole. Nevertheless, the high lipophilicity of **C8** should be an interesting option for its prospective clinical development, considering the possibility of local administration (intra-articular infiltration).

The toxicity and anti-inflammatory activity of **C8** were evaluated in rabbit articular chondrocytes in primary culture. The toxicity of **C8** was assessed by MTT, which allows an evaluation of the cell number and/or metabolic activity in cells. **C8** (from 0.31 to 9.92 µM) did not decrease cell viability in culture medium supplemented with 0.1% BSA or 2% FCS but did (at 4.96 and 9.92 µM) in culture medium alone. This observation is probably due to the cells being weakened in the absence of BSA or FCS. We also observed, as expected, an increase in cell number and/or metabolic activity in response to IL-1β. This effect increases in the presence of **C8**, at non toxic doses, whatever the culture conditions. Thus, depending on the culture conditions or **C8** doses, **C8** increases or decreases cell number and/or metabolic activity. Moreover, **C8** from 0.31 µM inhibited IL-1β-induced secretion of PGE_2_ by chondrocytes, corresponding to half of the IC_50_ on human GIIAPLA_2_ activity evaluated *in vitro* by enzymatic assay. Therefore, **C8** could be a potent anti-inflammatory drug *in vivo*. However, **C8** did not inhibit IL-1β-induced NO secretion by chondrocytes cultured in DMEM alone or supplemented with 0.1% BSA and slightly inhibited IL-1β-stimulated NO secretion in DMEM supplemented with 2% FCS. These data suggest that the anti-inflammatory property of **C8** in chondrocytes mainly depends on its capacity to inhibit PLA_2_ activity.

COX-1 is involved in normal physiological functions, whereas COX-2 is involved in the inflammatory response. Anti-inflammatory drugs such as NSAIDs and selective COX-2 inhibitors, used to treat rheumatic disease, have severe side effects owing to impairment in the balance between COX-1 and COX-2 [4], [5], [6]. Interestingly, the present work shows that the potent PLA_2_ inhibitor **C8** decreases PGE_2_ production without impairing this balance. Consequently, **C8** could be a useful candidate in developing new anti-inflammatory drugs lacking the side effects observed with NSAIDs and selective COX-2 inhibitors.

In summary, we report on the design, synthesis and testing of 7 **C1** analogs that differ from **C1** by indole substitution or by indole replacement by other aromatic rings, the largest being phenanthrene. Compounds **C2-C8** show both inhibitory activity on secreted/synovial GIIAPLA_2_ and selectivity as compared with GIBPLA_2_, a pancreatic enzyme involved in the digestion of dietary phospholipids. The order of interaction energies predicted by molecular modeling of these compounds is associated with their experimental IC_50_ values with GIIAPLA_2_ used as a target. The most promising compound is **C8** in terms of computed energy balance for binding GIIAPLA_2_ and experimental potency towards GIIAPLA_2_, namely one order of magnitude larger than that of **C1**. In addition, **C8** is endowed with anti-inflammatory activity in articular chondrocytes by inhibiting IL-1β-stimulated PGE_2_ secretion in these cells. Furthermore, it does not modify the ratio between the COX-1 and COX-2 isoenzymes. **C8** is therefore an attractive candidate for anti-inflammatory therapy in joints. Experiments in animal models of rheumatic diseases are in progress in our laboratory.

## Materials and Methods

### Ethics Statements

Experimental protocols using rabbits complied with French legislation on animal experimentation and were approved by INSERM (Intitut National de la Santé et de la Recherche Médicale)'s Committee for Animal Studies.

### Molecular modeling

Molecular modeling is described in Supporting Information S1.

### Synthesis of oxadiazolone derivatives

Synthesis of compounds **C1-C8** is described in Supporting Information S1.

### *In vitro* PLA_2_ assay

Fatty-acid free BSA and pancreatic PLA_2_ were from Sigma. hGIIAPLA_2_ was prepared as previously described [33]. The fluorescent substrate for PLA_2_ assay, 1-hexadecanoyl-2-(10-pyrenedecanoyl)-*sn*-glycero-3-phosphoglycerol, ammonium salt (β-py-C_10_-PG) was from Molecular Probes (Eugene). PLA_2_ activity was evaluated as previously described [34] with β-py-C_10_-PG used as a substrate (2 µM final concentration). In a total volume of 1 mL, the standard reaction medium contained 50 mM Tris-HCl (pH 7.5), 500 mM NaCl, 1 mM EGTA, 2 µM β-py-C_10_-PG, 0.1% fatty-acid free BSA and 6 ng/mL pancreatic PLA_2_ or 1 ng/mL hGIIAPLA_2_. The fluorescence (λ_ex_  = 342 nm and λ_em_  = 398 nm) of the enzymatic reaction medium was recorded for 3 min with use of a spectrofluorimeter LS 50 (Perkin-Elmer) equipped with a Xenon lamp. The reaction was initiated by the addition of CaCl_2_ (10 mM, final concentration). The increase in fluorescence was continuously recorded for 1 min, and PLA_2_ activity was calculated as previously described [34]. When used, the inhibitor was added to the reaction medium after introduction of BSA. The activity is expressed in micromoles of fluorescent β-py-C_10_-PG hydrolyzed per min. The standard error of the mean of three independent experiments was less than 10%, which allows for the determination of the IC_50_ values (concentration of inhibitors producing 50% inhibition) of each compound.

### Isolation and culture of chondrocytes from rabbit articular cartilage

Articular chondrocytes were isolated from 5-week-old Fauve de Bourgogne female rabbits (CPA, Orleans, France) and cultured at the first passage in conditions avoiding cell dedifferentiation as previously described [35]. Cells were cultured at 37°C in 12-well plates in Ham's F-12 medium containing 10% FCS, 20 IU/mL penicillin, and 20 µg/mL streptomycin (all from Invitrogen) until nearly confluent. Then medium was replaced with DMEM (Invitrogen) containing 20 IU/mL penicillin, and 20 µg/mL streptomycin and, if necessary, 0.1% fatty acid free BSA (Sigma) or 2% FCS. At this time the **C8** compound dissolved in DMSO (Sigma) was added to the medium (the amount of DMSO was kept at 1‰ (v/v) in all the wells). 1 h after the addition of **C8**, IL-1β (PeproTech) was added to the medium. Consequently, chondrocytes were incubated for 20 h with IL-1β and for 21 h with **C8**.

### Evaluation of cell viability

At 18 h after the addition of IL-1β, 3-[4,5-dimethylthiazol-2-yl]-2,5-diphenyl tetrazolium bromide (MTT; Sigma) was added to the cell culture medium at 0.5 mg/mL. Cells were incubated 2 more hours at 37°C. The medium was then removed, and DMSO was added to dissolve the formazan crystals. The absorbance of the resulting solution was spectrophotometrically measured at 570 and 690 nm (background). The value corresponding to absorbance_570nm_ - absorbance_690nm_ was directly proportional to the number and activity of the viable cells.

### Determination of PGE_2_ and nitrite concentrations in culture medium

20 h after the addition of IL-1β to the chondrocytes, culture media were collected, and aliquots were stored at −80°C until PGE_2_ and nitrite quantification. PGE_2_ concentration in culture media was determined by use of an enzyme immunoassay (EIA) kit (PGE_2_ EIA Kit-monoclonal; Cayman Chemical). Nitrite concentration was determined by a spectrophotometry method based on the Griess reaction [36]. Briefly, 200 µL of culture medium or sodium nitrite (NaNO_2_, Merck) standard dilutions were mixed with 100 µL Griess reagent [0.5% (w/v) sulphanilic acid (Merck), 0.05% (w/v) N(1-naphtyl)ethylenediamine (Merck), 30% (v/v) acetic acid, 1.5 N HCl] and incubated for 10 min at 50°C. The absorbance was measured at 540 nm.

### Preparation of whole-cell protein extracts, protein quantification and western blot analysis

Proteins were extracted from the cultured cells by addition of lysis buffer [10 mM Tris (pH 7.4), 0.5% (v/v) NP40, 150 mM NaCl, 1 mM PMSF, 0.1 mM Na_3_VO_3_, complete-EDTA-free protease inhibitor cocktail (Roche)]. Cell lysates were centrifuged for 15 min at 14000 rpm at 4°C and supernatants were collected. Protein concentrations were determined by the Bradford method [37] by use of the Protein Assay dye reagent (Bio-Rad). Protein extracts (20 µg) were size-separated by SDS-PAGE in a 10% (w/v) polyacrylamide gel and electroblotted to a nitrocellulose membrane. Equal protein loading and transfer was confirmed by staining the membrane with Ponceau Red [0.2% (w/v) in H_2_O:acetic acid 99∶1]. The membrane was sequentially incubated with antibodies against COX-1 (1∶200, Santa Cruz Biotechnology), COX-2 (1∶500, Santa Cruz Biotechnology), iNOS (1∶400, BD Biosciences) or α-tubulin (1∶100, Santa Cruz Biotechnology) and then with peroxidase-conjugated donkey anti-goat IgG (1∶20000) or donkey anti-rabbit IgG (1∶200, both Santa Cruz Biotechnology). Immunocomplexes were detected by an enhanced chemiluminescence kit (Amersham Bioscience). The membrane was stripped by incubation in 0.2 M NaOH between successive immunodetections. Semi-quantitative scanning densitometry involved use of the ImageJ program (NIH, USA).

### Statistical analysis

Results are expressed as means ± SEM for the number of experiments indicated. Statistical analysis involved use of the Kruskal-Wallis test, then the ANOVA Fisher's test. A *P*<0.05 was considered statistically significant.

## Supporting Information

Supporting Information S1 Materials and Methods in chemistry and molecular modeling.
(0.12 MB DOC)
