# Supplementary material for: Design of Group IIA Secreted/Synovial Phospholipase A2 Inhibitors: An Oxadiazolone Derivative Suppresses Chondrocyte Prostaglandin E2 Secretion
Source: PLoS One. 2010 Jun 1;5(6):e10914. doi: 10.1371/journal.pone.0010914 (PMC2879362; doi:10.1371/journal.pone.0010914)
Supplement: Supporting Information S1 — Materials and Methods in chemistry and molecular modeling. (0.12 MB DOC) [file pone.0010914.s001.doc]

**Supporting Information**

**Design of Group IIA Secreted/SYNOVIAL Phospholipase A_2_ inhibitors: an Oxadiazolone Derivative Suppresses Chondrocyte Prostaglandin E_2_ Secretion**

**Jean-Edouard Ombetta**^*^**^†^, Natacha Thelier^‡†^, Chang Zhi Dong**^§^**, Stéphanie Plocki**^§^**, Lydia Tsagris^‡^, François Rannou^‡¶^, France Massicot^||^, Atimé Djimdé**^§^**, Elissar El-Hayek^‡^, Yiming Shi**^§^**, Françoise Heymans**^§^**, Nohad Gresh^**^ and Caroline Chauvet^‡^.**

**Molecular modeling**

Molecular dynamics involved use of the DISCOVER (Accelrys) software (Accelrys Inc., San Diego, CA, USA). We use the 2 Angstroms resolution x-ray crystal structure of hsnp-PLA_2_ complexed with micromolar inhibitor OAP [1] (PDB access code 1kvo). The computations were carried out on the 70 N-terminal residues of PLA_2_. Restriction to these residues was supported by the x-ray structure showing all drug–protein interactions to take place exclusively on the N terminus. The ligands were constructed with the Builder module of the Accelrys package, with the Cff91 force-field. To derive the atomic charges on the oxadiazolone fragment in its anionic state, a Mulliken population analysis was performed following an *ab initio* Hartree-Fock computation with the 6-311G** basis set and the Gaussian 98 package [2]. Energy minimization and molecular dynamics were done in the absence of explicit water molecules (epsilon = 4). Throughout the simulations, the protein backbone was held frozen, and the side-chains, the Ca(II) cation, and the inhibitor were relaxed. Manual docking followed by a preliminary round of energy minimization involved use of our computer graphics facilities prior to molecular dynamics. The latter involved the same protocol as described [3]. The search for the best conformation of the uncomplexed protein and inhibitors followed the same molecular dynamics protocol. Solvation energies of the selected minima were computed by the Poisson-Boltzmann procedure with the Delphi software [4]. The solute and solvent dielectric constants were 4 and 80, respectively.

**General methods in chemistry**

All materials were obtained from commercial suppliers and used without further purification. Thin-layer chromatography was performed on TLC plastic sheets of silica gel 60F254 (layer thickness 0.2 mm) from Merck. Column chromatography purification was carried out on silica gel 60 (70-230 mesh ASTM, Merck). All melting points were determined on a digital melting point apparatus (Electrothermal) and are uncorrected. IR and 1H NMR spectra confirmed the structures of all compounds. IR spectra were obtained in paraffin oil with use of an ATI Mattson Genesis Series FTIR spectrometer, and 1H NMR spectra were recorded in CDCl3 on a BRUKER AC 200 spectrometer with hexamethyldisiloxane (HMDS) used as an internal standard. Chemical shifts are given in ppm and peak multiplicities are designated as follows: s, singlet; d, doublet; t, triplet; q, quintet; m, multiplet. Elemental analyses were obtained from the “Service régional de microanalyse” (Université Paris 6-Pierre et Marie Curie), Paris France, and were within ± 0.4% of theoretical values.

**Synthesis of oxadiazolone derivatives**

**4-(5-Bromopent-1-yloxy)benzyl cyanide** (**1**). 4-Hydroxybenzyl cyanide (3.5 g, 26.3 mmol), K_2_CO_3_ (4.75 g, 34.4 mmol) and 1,5-dibromopentane (6.5 mL, 47.7 mmol) in DMF (50 mL) were stirred at room temperature for 10 days. The solvent was then removed under reduced pressure, and the residue was taken up in EtOAc, washed with H_2_O to neutral pH, dried over MgSO_4_, and filtered. The filtrate was condensed to dryness, and the residue was purified by silica gel column chromatography with ether/petroleum ether (15 : 85, v/v) used as an eluent to give the title compound (4.45 g, 60% yield) as a yellow oil: IR (nujol, cm^-1^) 2249 (CN), 1613 (C=C_ar_); ^1^H NMR (200 MHz, CDCl_3_) δ 1.51-1.91 (m, 6H, OCH_2_(CH_2_)_3_), 3.37 (t, J = 6.68, 2H, CH_2_Br), 3.60 (s, 2H, CH_2_CN), 3.90 (t, J = 6.18, 2H, CH_2_O), 6.82 (d, J = 8.56, 2H, H_ar_), 7.15 (d, J = 8.56, 2H, H_ar_); ^13^C NMR (50 MHz, CDCl_3_) δ 22.64 (CH_2al_), 24.63 (CH_2al_), 28.20 (CH_2al_), 32.28 (CH_2al_), 33.50 (CH_2_Br), 67.54 (OCH_2_), 114.90 (CH_ar_), 118.13 (CN), 121.60 (Cq_ar_), 128.93 (CH_ar_), 158.56 (O-Cq_ar_).

General procedure to synthesize **2a-b, e-g**: to a solution of an aromatic alcohol (1.2 eq) in EtOH (2 mL/mmol) at 0 °C was added a solution of NaOH (1.2 eq) dropwise in the same solvent (1 mL/mmol). After being stirred for 15 min, the solvent was eliminated under reduced pressure, and the residue, dissolved in DMF (1.5 mL/mmol), was dropped into a solution of **1** (1 eq) in DMF (2 mL/mmol) at room temperature. The mixture was stirred at the same temperature overnight and then evaporated to dryness in vacuum. The remaining slurry oil was taken up into CH_2_Cl_2_, washed with H_2_O, dried over MgSO_4_ and then filtered. Silica gel column chromatography or crystallization was performed to obtain the pure compounds **2a-b, e-g**.

**4-(5-(*o*-Phenylphenoxy)pent-1-yloxy)phenylacetonitrile** (**2a**) was obtained following the general procedure from 2-phenylphenol in 71% yield as a yellow oil after purification by chromatography (CH_2_Cl_2_/PE, 50:50, v/v): IR (neat, cm^-1^) 2249 (CN), 1612 (C=C_ar_); ^1^H NMR (200 MHz, CDCl_3_) δ 1.45 (m, 2H, OCH_2_CH_2_CH_2_), 1.64 (m, 4H, OCH_2_CH_2_), 3.52 (s, 2H, CH_2_CN), 3.77 (t, J = 6.30, 2H, OCH_2_), 3.87 (t, J = 6.10, 2H, OCH_2_), 6.75 (d, J = 8.51, 2H, H_ar_), 6.90 (m, 2H, H_ar_), 7.09 (d, J = 8.51, 2H, H_ar_), 7.23 (m, 5H, H_ar_), 7.44 (m, 2H, H_ar_); ^13^C NMR (50 MHz, CDCl_3_) δ 22.59 (CH_2al_), 22.63 (CH_2al_), 28.65 (CH_2al_), 28.74 (CH_2_CN), 67.72 (OCH_2_), 68.04 (OCH_2_), 112.46 (CH_ar_), 114.93 (CH_ar_), 118.17 (CN), 120.77 (CH_ar_), 121.51 (Cq_ar_), 126.62 (CH_ar_), 127.70 (CH_ar_), 128.47 (CH_ar_), 128.93 (CH_ar_), 129.50 (CH_ar_), 130.74 (CH_ar_), 130.84 (Cq_ar_), 138.50 (Cq_ar_), 155.80 (O-Cq_ar_), 158.69 (O-Cq_ar_).

**4-(5-(2-Methylbenzothiazol-5-yloxy)pent-1-yloxy)phenylacetonitrile** (**2b**) was obtained following the general procedure from 2-methyl-5-benzothiazolol in 57% yield as a yellow solid after purification by chromatography (CH_2_Cl_2_/PE, 60:40, v/v): mp < 30 °C; IR (nujol, cm^-1^) 2249 (CN), 1604 (C=C_ar_); ^1^H NMR (200 MHz, CDCl_3_) δ 1.58 (m, 2H, OCH_2_CH_2_CH_2_), 1.78 (m, 4H, OCH_2_CH_2_), 2.72 (s, 3H, CH_3_), 3.57 (s, 2H, CH_2_CN), 3.90 (t, J = 6.32, 2H, OCH_2_), 3.97 (t, J = 6.24, 2H, OCH_2_), 6.80 (d, J = 8.54, 2H, H_ar_), 6.90 (dd, J_1_ = 8.76, J_2_ = 2.40, 1H, H_ar_), 7.12 (d, J = 8.54, 2H, H_ar_), 7.36 (d, J = 2.40, 1H, H_ar_), 7.56 (d, J = 8.75, 1H, H_ar_); ^13^C NMR (50 MHz, CDCl_3_) δ 20.01 (CH_3_), 22.62 (CH_2al_), 28.80 (CH_2_CN), 67.70 (OCH_2_), 67.96 (OCH_2_), 105.76 (CH_ar_), 114.84 (CH_ar_), 114.91 (CH_ar_), 118.12 (CN), 121.44 (CH_ar_), 121.51 (Cq_ar_), 128.90 (CH_ar_), 154.45 (Cq_ar_), 158.06 (Cq_ar_), 158.64 (Cq_ar_), 168.02 (Cq_ar_).

**4-(5-(*p*-Phenylphenoxy)pent-1-yloxy)phenylacetonitrile** (**2e**) was obtained in 57% yield as yellow crystals following the general procedure from 4-phenylphenol after 5 days of reaction and crystallization (EtOH): mp 90 °C; IR (nujol, cm^-1^) 2253 (CN), 1610 (C=C_ar_); ^1^H NMR (200 MHz, CDCl_3_) δ 1.61 (m, 2H, OCH_2_CH_2_CH_2_), 1.81 (m, 4H, OCH_2_CH_2_), 3.59 (s, 2H, CH_2_CN), 3.91 (t, J = 6.25, 2H, OCH_2_), 3.96 (t, J = 6.25, 2H, OCH_2_), 6.82 (d, J = 8.54, 2H, H_ar_), 6.89 (d, J = 8.59, 2H, H_ar_), 7.14 (d, J = 8.54, 2H, H_ar_), 7.38 (m, 7H, H_ar_); ^13^C NMR (50 MHz, CDCl_3_) δ 22.70 (CH_2al_), 22.77 (CH_2al_), 28.92 (CH_2al_), 28.99 (CH_2_CN), 67.75 (OCH_2_), 67.83 (OCH_2_), 114.73 (CH_ar_), 115.04 (CH_ar_), 118.19 (CN), 121.60 (Cq_ar_), 126.60 (CH_ar_), 126.66 (CH_ar_), 128.08 (CH_ar_), 128.67 (CH_ar_), 129.02 (CH_ar_), 133.61 (Cq_ar_), 140.78 (Cq_ar_), 158.55 (O-Cq_ar_), 158.77 (O-Cq_ar_).

**4-(5-(Phenoxy)pent-1-yloxy)phenylacetonitrile** (**2f**) was obtained following the general procedure from phenol in 25% yield as a yellow oil after purification by chromatography (CH_2_Cl_2_/PE, 40:60, v/v): IR (neat, cm^-1^) 2249 (CN), 1599 (C=C_ar_); ^1^H NMR (200 MHz, CDCl_3_) δ 1.58 (m, 2H, OCH_2_CH_2_CH_2_), 1.78 (m, 4H, OCH_2_CH_2_), 3.57 (s, 2H, CH_2_CN), 3.89 (t, J = 6.27, 2H, OCH_2_), 3.90 (t, J = 6.19, 2H, OCH_2_), 6.83 (m, 5H, H_ar_), 7.17 (m, 4H, H_ar_); ^13^C NMR (50 MHz, CDCl_3_) δ 22.63 (CH_2al_), 22.67 (CH_2al_), 28.86 (CH_2al_), 28.93 (CH_2_CN), 67.47 (OCH_2_), 67.78 (OCH_2_), 114.38 (CH_ar_), 114.97 (CH_ar_), 118.17 (CN), 120.48 (CH_ar_), 121.56 (Cq_ar_), 128.96 (CH_ar_), 129.34 (CH_ar_), 158.71 (O-Cq_ar_), 158.91 (O-Cq_ar_).

**4-(5-(9-Phenanthryloxy)pent-1-yloxy]phenylacetonitrile** (**2g**) was obtained following the general procedure from 9-phenanthrol in 31% yield as a red oil after 3 days of reaction and purification by chromatography (CH_2_Cl_2_/PE, 25:75 to 40:60, v/v): IR (neat, cm^-1^) 2249 (CN), 1595 (C=C_ar_); ^1^H NMR (200 MHz, CDCl_3_) δ 1.79 (m, 6H, OCH_2_(CH_2_)_3_), 3.53 (s, 2H, CH_2_CN), 3.89 (t, J = 6.00, 2H, OCH_2_), 4.13 (t, 2H, J = 6.20, OCH_2_), 6.78 (m, 3H, H_ar_), 7.08 (d, J = 8.47, 2H, H_ar_), 7.55 (m, 5H, H_ar_), 8.30 (m, 1H, H_ar_), 8.53 (m, 2H, H_ar_); ^13^C NMR (50 MHz, CDCl_3_) δ 22.66 (CH_2al_), 22.83 (CH_2al_), 28.24 (CH_2al_), 29.05 (CH_2_CN), 67.59 (OCH_2_), 67.77 (OCH_2_), 102.49 (CH_ar_), 114.98 (CH_ar_), 118.16 (CN), 121.55 (Cq_ar_), 122.40 (CH_ar_), 122.96 (CH_ar_), 123.79 (CH_ar_), 124.07 (CH_ar_), 126.33 (CH_ar_), 128.93 (CH_ar_), 129.50 (CH_ar_), 130.81 (CH_ar_), 130.84 (Cq_ar_), 130.84 (Cq_ar_), 132.87 (Cq_ar_), 135.82 (CH_ar_), 152.68 (O-Cq_ar_), 158.69 (O-Cq_ar_), 180.10 (Cq_ar_).

General procedure to synthesize **2c-d**: the aromatic amine (1.2 eq), **1** (1 eq) and K_2_CO_3_ (1.5 eq) in CH_3_CN (5 mL/mmol) were stirred at room temperature for the times indicated for each compound. The mixture was then filtered and the filtrate condensed to dryness. The residue dissolved in EtOAc was washed with H_2_O to neutral pH and brine, dried over MgSO_4_ and filtered. The solvent was removed under reduced pressure, and the crude product was purified by silica gel column chromatography with CH_2_Cl_2_/petroleum ether (PE) to provide the pure **2c-d**.

**4-(5-(5-Chloroindol-1-yl)pent-1-yloxy)phenylacetonitrile** (**2c**) was prepared according to the general procedure from 5-chloroindole in 30% yield as a green oil after 5 days of reaction and purification (CH_2_Cl_2_/PE, 30:70, v/v): IR (neat, cm^-1^) 2249 (CN), 1612 (C=C_ar_); ^1^H NMR (200 MHz, CDCl_3_) δ 1.40 (m, 2H, OCH_2_CH_2_CH_2_), 1.82 (m, 4H, NCH_2_CH_2_, OCH_2_CH_2_), 3.56 (s, 2H, CH_2_CN), 3.80 (t, J = 6.26, 2H, CH_2_O), 4.02 (t, J = 6.98, 2H, CH_2_N), 6.34 (dd, J_1_ = 3.12, J_2_ = 0.72, 1H, H_ar_), 6.75 (d, J = 8.73, 2H, H_ar_), 7.08 (m, 5H, H_ar_), 7.50 (dd, J_1_ = 1.95, J_2_ = 0.44, 1H, H_ar_); ^13^C NMR (50 MHz, CDCl_3_) δ 22.69 (CH_2al_), 23,43 (CH_2al_), 28,69 (CH_2al_), 29,85 (CH_2_CN), 46,34 (CH_2_N), 67,53 (CH_2_O), 100,63 (CH_ar_), 110,26 (CH_ar_), 114,94 (CH_ar_φo), 118,17 (CN),120,20 (CH_ar_), 121,57 (CH_ar_), 121,66 (Cq_ar_), 124,87 (Cq_ar_), 128,99 (CH_ar_φm), 134,27 (Cq_ar_), 158,58 (O-Cq_ar_).

**4-(5-(5-Methoxyindol-1-yl)pent-1-yloxy)phenylacetonitrile** (**2d**) was prepared according to the general procedure from 5-methoxyindole in 53% yield as a yellowish green oil after 7 days of reaction and purification (CH_2_Cl_2_/PE, 25:75, v/v): IR (neat, cm^-1^) 2248 (CN), 1614 (C=C_ar_); ^1^H NMR (200 MHz, CDCl_3_) δ 1.39 (m, 2H, OCH_2_CH_2_CH_2_), 1.73 (m, 4H, NCH_2_CH_2_, OCH_2_CH_2_), 3.54 (s, 2H, CH_2_CN), 3.76 (s, 3H, OCH_3_), 3.80 (t, J = 6.28, 2H, CH_2_O), 4.00 (t, J = 6.92, 2H, CH_2_N), 6.32 (d, J = 3.00, 1H, H_ar_), 6.77 (m, 3H, H_ar_), 6.99 (m, 2H, H_ar_), 7.12 (m, 3H, H_ar_); ^13^C NMR (50 MHz, CDCl_3_) δ 22.69 (CH_2al_), 23.43 (CH_2al_), 28.69 (CH_2al_), 29.85 (CH_2_CN), 46.34 (CH_2_N), 55.73 (OCH_3_), 67.53 (CH_2_O), 100.38 (CH_ar_), 102.45 (CH_ar_), 109.95 (CH_ar_), 111.65 (CH_ar_), 114.91 (CH_ar_φo), 118.17 (CN), 121.63 (Cq_ar_), 128.20 (CH_ar_), 124.79 (Cq_ar_), 128.94 (CH_ar_φm), 131.72 (Cq_ar_), 153.80 (O-Cq_ar_), 158.58 (O-Cq_ar_).

General procedure to prepare the amidoximes **3a-g**: each of the nitriles **2a-g** (1 eq), hydroxylamine hydrochloride (5 eq) and K_2_CO_3_ (5.5 eq) were heated to reflux of absolute ethanol (10 mL/mmol) for 24 h. The salts were filtered and the filtrate was evaporated under reduced pressure. The residue was then directly purified by silica gel column chromatography to produce the pure corresponding amidoxime.

***N*-Hydroxy-4-(5-(*o*-phenylphenoxy)pent-1-yloxy)phenylacetamidine** (**3a**) was synthesized according to the general procedure from **2a** (0.65 g, 1.75 mmol) in 80% yield as a yellow oil after purification (MeOH/CH_2_Cl_2_, 2:98, v/v): IR (neat, cm^-1^) 3508, 3401 (NH_2_), 3263 (OH), 1665 (C=N), 1611 (C=C_ar_); ^1^H NMR (200 MHz, CDCl_3_) δ 1.46 (m, 2H, OCH_2_CH_2_CH_2_), 1.68 (m, 4H, OCH_2_CH_2_), 3.32 (s, 2H, CH_2_C=N), 3.80 (t, J = 6.31, 2H, CH_2_O), 3.89 (t, J = 6.13, 2H, CH_2_O), 4.42 (s, 2H, NH_2_), 6.74 (d, J = 8.61, 2H, H_ar_), 6.90 (m, 2H, H_ar_), 7.09 (d, J = 8.61, 2H, H_ar_), 7.23 (m, 5H, H_ar_), 7.46 (m, 2H, H_ar_); ^13^C NMR (50 MHz, CDCl_3_) δ 22.65 (CH_2al_), 28.80 (CH_2al_), 36.69 (CH_2_C=N), 67.69 (CH_2_O), 68.13 (CH_2_O), 112.51 (CH_ar_), 114.72 (CH_ar_), 120.80 (CH_ar_), 126.69 (CH_ar_), 127.56 (Cq_ar_), 127.74 (CH_ar_), 128.49 (CH_ar_), 129.54 (CH_ar_), 129.86 (CH_ar_), 130.92 (Cq_ar_), 138.54 (Cq_ar_), 153.27 (C=N), 155.86 (O-Cq_ar_), 158.19 (O-Cq_ar_).

***N*-Hydroxy-4-(5-(2-methylbenzothiazol-5-yloxy)pent-1-yloxy)phenylacetamidine** (**3b**) was synthesized according to the general procedure from **2b** (0.70 g, 1.91 mmol) in 53% yield as a green oil after purification (MeOH/CH_2_Cl_2_, 0.5:99.5 to 1:99, v/v): IR (neat, cm^-1^) 3472, 3374 (NH_2_), 3150 (OH), 1655 (C=N), 1581 (C=C_ar_); ^1^H NMR (200 MHz, CDCl_3_/CD_3_OD) δ 1.62 (m, 2H, OCH_2_CH_2_CH_2_), 1.79 (m, 4H, OCH_2_CH_2_), 2.74 (s, 3H, CH_3_), 3.31 (s, 2H, CH_2_C=N), 3.91 (t, J = 6.20, 2H, OCH_2_), 3.98 (t, J = 6.23, 2H, OCH_2_), 4.55 (br s, 2H, NH_2_), 6.78 (d, J = 8.42, 2H, H_ar_), 6.92 (dd, J_1_ = 8.76, J_2_ = 2.33, 1H, H_ar_), 7.12 (d, J = 8.42, 2H, H_ar_), 7.36 (d, J = 2.33, 1H, H_ar_), 7.56 (d, J = 8.75, 1H, H_ar_); ^13^C NMR (50 MHz, CDCl_3_/CD_3_OD) δ 19.86 (CH_3_), 22.62 (CH_2al_), 28.82 (CH_2al_), 28.87 (CH_2al_), 36.48 (CH_2_C=N), 67.69 (CH_2_O), 68.07 (CH_2_O), 105.62 (CH_ar_), 114.72 (CH_ar_), 115.02 (CH_ar_), 121.50 (CH_ar_), 126.95 (Cq_ar_), 127.50 (Cq_ar_), 129.72 (CH_ar_), 153.54 (Cq_ar_), 154.17 (C=N), 158.13 (Cq_ar_), 158.17 (Cq_ar_), 168.52 (Cq_ar_).

***N*-Hydroxy-4-(5-(5-chloroindol-1-yl)pent-1-yloxy)phenylacetamidine** (**3c**) was synthesized according to the general procedure from **2c** (1.00 g, 2,9 mmol) in 36% yield as a yellow oil after purification (MeOH/CH_2_Cl_2_, 1:99, v/v): IR (neat, cm^-1^) 3481, 3379 (NH_2_), 3180 (OH), 1663 (C=N), 1611 (C=C_ar_); ^1^H NMR (200 MHz, CDCl_3_) δ 1.40 (m, 2H, OCH_2_CH_2_CH_2_), 1.76 (m, 4H, NCH_2_CH_2_, OCH_2_CH_2_), 3.31 (s, 2H, CH_2_C=N), 3.79 (t, J = 6.24, 2H, CH_2_O), 4.02 (t, J = 6.94, 2H, CH_2_N), 4.43 (br s, 2H, NH_2_), 6.34 (d, J = 3.05, 1H, H_ar_), 6.72 (dd, J_1_ = 8.60, J_2_ = 1.89, 2H, H_ar_), 7.07 (m, 5H, H_ar_), 7.07 (br s, 1H, OH), 7.50 (d, J = 1.80, 1H, H_ar_); ^13^C NMR (50 MHz, CDCl_3_) δ 23.28 (CH_2al_), 28.53 (CH_2al_), 29.28 (CH_2al_), 36.58 (CH_2_C=N), 47.93 (CH_2_N), 67.33 (CH_2_O), 100.63 (CH_ar_), 110.27 (CH_ar_), 114.65 (CH_ar_φo), 120.21 (CH_ar_), 123.81 (CH_ar_), 121.58 (CH_ar_), 124.87 (Cq_ar_), 127.69 (Cq_ar_), 129.47 (CH_ar_φm), 134.27 (Cq_ar_), 153.26 (C=N), 158.03 (O-Cq_ar_).

***N*-Hydroxy-4-(5-(5-methoxyindol-1-yl)pent-1-yloxy)phenylacetamidine** (**3d**) was synthesized according to the general procedure from **2d** (0.45 g, 1.68 mmol) in 61% yield as a yellow oil after purification (MeOH/CH_2_Cl_2_, 2:98, v/v): IR (neat, cm^-1^) 3480, 3360 (NH_2_), 3195 (OH), 1664 (C=N), 1612 (C=C_ar_); ^1^H NMR (200 MHz, CDCl_3_) δ 1.40 (m, 2H, OCH_2_CH_2_CH_2_), 1.77 (m, 4H, NCH_2_CH_2_, OCH_2_CH_2_), 3.31 (s, 2H, CH_2_C=N), 3.77 (s, 3H, OCH_3_), 3.81 (t, J = 6.24, 2H, CH_2_O), 4.02 (t, J = 6,91, 2H, CH_2_N), 4.42 (br s, 2H, NH_2_), 6.32 (d, J = 3.04, 1H, H_ar_), 6.50 (br s, 1H, OH), 6.75 (m, 3H, H_ar_), 7.06 (m, 5H, H_ar_); ^13^C NMR (50 MHz, CDCl_3_) δ 23.53 (CH_2al_), 28.81 (CH_2al_), 29.99 (CH_2al_), 36.68 (CH_2_C=N), 46.38 (CH_2_N), 55.82 (OCH_3_), 67.55 (CH_2_O), 100.43 (CH_ar_), 102.52 (CHar), 110.01 (CH_ar_), 111.73 (CH_ar_), 114.73 (CH_ar_), 127.59 (Cq_ar_), 128.26 (CH_ar_), 128.84 (Cq_ar_), 129.87 (CH_ar_), 131.24 (Cq_ar_), 153.24 (Cq_ar_), 153.85 (C=N), 158,12 (O-Cq_ar_).

***N*-Hydroxy-4-(5-(*p*-phenylphenoxy)pent-1-yloxy)phenylacetamidine** (**3e**) was synthesized according to the general procedure from **2e** (0.51 g, 1.37 mmol) in 35% yield as a white solid after purification (MeOH/CH_2_Cl_2_, 1:99 to 2 :98, v/v): mp 139.3 °C; IR (nujol, cm^-1^) 3424, 3312 (NH_2_), 3244 (OH), 1659 (C=N), 1605 (C=C_ar_); ^1^H NMR (200 MHz, CDCl_3_) δ 1.62 (m, 2H, OCH_2_CH_2_CH_2_), 1.78 (m, 4H, OCH_2_CH_2_), 3.31 (s, 2H, CH_2_C=N), 3.91 (t, J = 6.34, 2H, CH_2_O), 3.96 (t, J = 6.12, 2H, CH_2_O), 4.58 (br s, 2H, NH_2_), 6.79 (d, J = 8.45, 2H, H_ar_), 6.89 (d, J = 8.59, 2H, H_ar_), 7.10 (d, J = 8.45, 2H, H_ar_), 7.38 (m, 7H, H_ar_); ^13^C NMR (50 MHz, CDCl_3_) δ 22.62 (CH_2al_), 28.91 (CH_2al_), 36.49 (CH_2_C=N), 67.73 (CH_2_O), 114.67 (CH_ar_), 114.73 (CH_ar_), 126.57 (CH_ar_), 127.50 (Cq_ar_), 128.00 (CH_ar_), 128.60 (CH_ar_), 129.74 (CH_ar_), 133.55 (Cq_ar_), 140.70 (Cq_ar_), 153.56 (C=N), 158.16 (O-Cq_ar_), 158.47 (O-Cq_ar_).

***N*-Hydroxy-4-(5-(phenoxy)pent-1-yloxy)phenylacetamidine** (**3f**) was synthesized according to the general procedure from **2f** (0.14 g, 0.48 mmol) in 74% yield as a yellow solid after purification (MeOH/CH_2_Cl_2_, 1:99 to 2 :98, v/v): mp 103.2 °C; IR (nujol, cm^-1^) 3487, 3395 (NH_2_), 3194 (OH), 1654 (C=N), 1583 (C=C_ar_); ^1^H NMR (200 MHz, CDCl_3_) δ 1.59 (m, 2H, OCH_2_CH_2_CH_2_), 1.78 (m, 4H, OCH_2_CH_2_), 3.33 (s, 2H, CH_2_C=N), 3.90 (m, 4H, CH_2_O), 4.44 (s, 2H, NH_2_), 6.83 (m, 5H, H_ar_), 7.17 (m, 4H, H_ar_); ^13^C NMR (50 MHz, CDCl_3_) δ 22.69 (CH_2al_), 28.98 (CH_2al_), 36.70 (CH_2_C=N), 67.55 (CH_2_O), 67.75 (CH_2_O), 114.43 (CH_ar_), 114.76 (CH_ar_), 120.52 (CH_ar_), 127.56 (Cq_ar_), 129.38 (CH_ar_), 129.88 (CH_ar_), 158.22 (C=N), 155.96 (O-Cq_ar_).

***N*-Hydroxy-4-(5-(9-phenanthryloxy)pent-1-yloxy)phenylacetamidine** (**3g**) was synthesized according to the general procedure from **2g** (0.30 g, 0.76 mmol) in 61% yield as a yellow oil after purification (MeOH/CH_2_Cl_2_, 1:99 to 2:98, v/v): IR (neat, cm^-1^) 3492, 3385 (NH_2_), 3195 (OH), 1661 (C=N), 1600 (C=C_ar_); ^1^H NMR (200 MHz, CDCl_3_) δ 1.88 (m, 6H, OCH_2_(CH_2_)_3_), 3.33 (s, 2H, CH_2_C=N), 3.94 (t, J = 5.85, 2H, CH_2_O), 4.19 (t, J = 6.14, 2H, CH_2_O), 4.44 (s, 2H, NH_2_), 6.83 (m, 3H, H_ar_), 7.10 (d, J = 8.47, 2H, H_ar_), 7.55 (m, 5H, H_ar_), 8.30 (m, 1H, H_ar_), 8.53 (m, 2H, H_ar_); ^13^C NMR (50 MHz, CDCl_3_) δ 22.79 (CH_2al_), 22.95 (CH_2al_), 28.98 (CH_2al_), 30.07 (CH_2al_), 36.68 (CH_2_C=N), 67.70 (CH_2_O), 67.78 (CH_2_O), 102.54 (CH_ar_), 114.83 (CH_ar_), 122.49 (CH_ar_), 123.05 (CH_ar_), 124.12 (CH_ar_), 126.30 (CH_ar_), 126.83 (CH_ar_), 127.07 (CH_ar_), 127.45 (Cq_ar_), 127.67 (CH_ar_), 129.91 (CH_ar_), 132.91 (Cq_ar_), 153.51 (C=N), 158.29 (O-Cq_ar_).

General procedure to prepare the final products **(C2-C8)**: to a solution of amidoxime (1 eq) in CH_2_Cl_2_ (10 mL/mmol) in the presence of Et_3_N (1.5 eq) at 0 °C was added phenyl chloroformate (1.1 eq). Upon completion, the solution was stirred for 1 h, then washed with water to neutral pH and dried over MgSO_4_. The solvent was removed under reduced pressure and the carbonate, dissolved in toluene, was heated to reflux for the time period indicated for each of the compounds. After evaporation of the solvent, the residue was purified by silica gel column chromatography to yield the pure oxadiazolone derivatives **(C2-C8)**.

**4,5-Dihydro-3-(4-(5-(o-phenylphenoxy)pent-1-yloxy)benzyl)-1,2,4-4H-oxadiazol-5-one** (**C2**) was obtained according to the general procedure from **3a** (0.56 g, 1.38 mmol) in 50% yield as a yellow oil after 4h of reflux and purification (CH_2_Cl_2_): IR (neat, cm^-1^) 3184 (NH), 1765 (C=O), 1612 (C=C_ar_); ^1^H RMN (200 MHz, CDCl_3_/CD_3_OD) δ 1.47 (m, 2H, OCH_2_CH_2_CH_2_), 1.67 (m, 4H, OCH_2_CH_2_), 3.69 (s, 2H, CH_2_C=N), 3.79 (t, J = 6.20, 2H, CH_2_O), 3.90 (t, J = 6.12, 2H, CH_2_O), 6.75 (d, J = 8.54, 2H, H_ar_), 6.92 (m, 2H, H_ar_), 7.08 (d, J = 8.54, 2H, H_ar_), 7.23 (m, 5H, H_ar_), 7.46 (m, 2H, H_ar_), 10.10 (s, 1H, NH); ^13^C RMN (50 MHz, CDCl_3_) δ 22.65 (CH_2al_), 28.73 (CH_2al_), 28.81 (CH_2al_), 30.65 (CH_2_C=N), 67.76 (CH_2_O), 68.15 (CH_2_O), 112.55 (CH_ar_), 115.14 (Cq_ar_), 120.84 (CH_ar_), 123.81 (Cq_ar_), 126.71 (CH_ar_), 127.77 (CH_ar_), 128.52 (CH_ar_), 12956 (CH_ar_), 129.90 (CH_ar_), 130.83 (CH_ar_), 130.95 (Cq_ar_), 138.56 (Cq_ar_), 155.87 (O-Cq_ar_), 158.52 (C=N), 158.87 (O-Cq_ar_), 161.19 (C=O). Anal. (C_26_H_26_N_2_O_4_∙1/4H_2_O) C, N, H.

**4,5-Dihydro-3-(4-(5-(2-methylbenzothiazol-5-yloxy)pent-1-yloxy)benzyl)-1,2,4-4H-oxadiazol-5-one** (**C3**) was obtained according to the general procedure from **3b** (0.40 g, 1.09 mmol) in 40% yield as a beige solid after 6h of reflux and purification (CH_2_Cl_2_ to MeOH/CH_2_Cl_2_, 2:98, v/v): mp 128.1 °C; IR (nujol, cm^-1^) 3146 (NH), 1773 (C=O), 1603 (C=C_ar_); ^1^H RMN (200 MHz, CDCl_3_/CD_3_OD) δ 1.57 (m, 2H, OCH_2_CH_2_CH_2_), 1.79 (m, 4H, OCH_2_CH_2_), 2.68 (s, 3H, CH_3_), 3.66 (s, 3H, CH_3_), 3.86 (t, J = 6.15, 2H, OCH_2_), 3.94 (t, J = 6.17, 2H, OCH_2_), 6.74 (d, J = 8.58, 2H, H_ar_), 6.92 (dd, J_1_ = 8.76, J_2_ = 2.34, 1H, H_ar_), 7.12 (d, J = 8.58, 2H, H_ar_), 7.36 (d, J = 2.34, 1H, H_ar_), 7.56 (d, J = 8.76, 1H, H_ar_); ^13^C RMN (50 MHz, CDCl_3_/CD_3_OD) δ 19.52 (CH_3_), 22.42 (CH_2al_), 28.63 (CH_2al_), 30.39 (CH_2_C=N), 67.56 (CH_2_O), 67.92 (CH_2_O), 105.33 (CH_ar_), 114.72 (CH_ar_φo), 114.85 (CH_ar_), 121.42 (CH_ar_), 124.31 (Cq_ar_), 126.69 (Cq_ar_), 129.55 (CH_ar_φm), 153.74 (Cq_ar_), 158.06 (Cq_ar_), 158.39 (Cq_ar_), 158.61 (C=N), 160.56 (C=O), 168.72 (Cq_ar_). Anal. (C_22_H_23_N_3_O_4_S∙1/6PhOH∙1/6EtOH) C, N, H.

**4,5-Dihydro-3-(4-(5-(5-chloroindol-1-yl)pent-1-yloxy)benzyl)-1,2,4-4H-oxadiazol-5-one** (**C4**) was obtained according to the general procedure from **3c** (0.70 g, 1.82 mmol) in 47% yield as a white solid after 4h of reflux and purification (EtOAc/CH_2_Cl_2_, 5:95, v/v): mp 121.5 °C ; IR (nujol, cm^-1^) 3200 (NH), 1769 (C=O), 1611 (C=C_ar_); ^1^H RMN (200 MHz, CD_3_OD/CDCl_3_) δ 1.39 (m, 2H, OCH_2_CH_2_CH_2_), 1.75 (m, 4H, NCH_2_CH_2_, OCH_2_CH_2_), 3.69 (s, 2H, CH_2_C=N), 3.81 (t, J = 6.28, 2H, CH_2_O), 4.04 (t, J = 6.91, 2H, CH_2_N), 6.34 (d, J = 3.10, 1H, H_ar_), 6.75 (d, J = 8.66, 2H, H_ar_), 7.10 (m, 5H, H_ar_), 7.50 (d, J = 1.92, 1H, H_ar_); ^13^C RMN (50 MHz, CDCl_3_/CD_3_OD) δ 23.37 (CH_2al_), 28.64 (CH_2al_), 29.80 (CH_2al_), 30.55 (CH_2_C=N), 46.28 (CH_2_N), 67.52 (CH_2_O), 100.54 (CH_ar_), 110.22 (CH_ar_), 114.86 (CH_ar_φo), 120.10 (CH_ar_), 121.49 (CH_ar_), 124.41 (Cq_ar_), 124.78 (Cq_ar_), 128.92 (CH_ar_), 129.41 (Cq_ar_), 129.74 (CH_ar_φm), 134.22 (Cq_ar_), 158.49 (O-Cq_ar_), 158.59 (C=N), 160.74 (C=O). Anal. (C_22_H_22_N_3_O_3_Cl) C, N, H.

**4,5-Dihydro-3-(4-(5-(5-methoxyindol-1-yl)pent-1-yloxy)benzyl)-1,2,4-4H-oxadiazol-5-one** (**C5**) was obtained according to the general procedure from **3d** (0.30 g, 0.78 mmol) in 34% yield as a red oil after 8h of reflux and purification (EtOAc/CH_2_Cl_2_, 2:98, v/v): IR (neat, cm^-1^) 3167 (NH), 1768 (C=O), 1612 (C=C_ar_); ^1^H RMN (200 MHz, CDCl_3_/CD_3_OD) δ 1.38 (m, 2H, OCH_2_CH_2_CH_2_), 1.75 (m, 4H, NCH_2_CH_2_, OCH_2_CH_2_), 3.66 (s, 2H, CH_2_C=N), 3.75 (s, 3H, OCH_3_), 3.78 (t, J = 6.28, 2H, CH_2_O), 4.00 (t, J = 6.91, 2H, CH_2_N), 6.31 (d, J = 3.02, 2H, H_ar_), 6.73 (m, 3H, H_ar_), 7.04 (m, 5H, H_ar_); ^13^C RMN (50 MHz, CDCl_3_/CD_3_OD) δ 23.44 (CH_2al_), 28.70 (CH_2al_), 29.89 (CH_2al_), 30.58 (CH_2_C=N), 46.34 (CH_2_N), 55.87 (OCH_3_), 67.54 (CH_2_O), 100.42 (CH_ar_), 102.60 (CH_ar_), 110.02 (CH_ar_), 111.70 (CH_ar_), 115.05 (CH_ar_), 123.96 (Cq_ar_), 128.30 (CH_ar_), 128.83 (Cq_ar_), 129.88 (CH_ar_), 131.24 (Cq_ar_), 153.75 (O-Cq_ar_), 158.54 (C=N), 158.68 (Cq_ar_), 161.03 (C=O). Anal. (C_23_H_25_N_3_O_4_∙1.1 EtOH) C, N, H.

**4,5-Dihydro-3-(4-(5-(p-phenylphenoxy)pent-1-yloxy)benzyl)-1,2,4-4H-oxadiazol-5-one** (**C6**) was obtained according to the general procedure from **3e** (0.20 g, 0.49 mmol) in 48% yield as a pink solid after 5h of reflux and purification (CH_2_Cl_2_): mp 151.9 °C; IR (nujol, cm^-1^) 3313 (NH), 1836 (C=O), 1727 (C=N), 1604 (C=C_ar_); ^1^H RMN (200 MHz, CD_3_OD/CDCl_3_) δ 1.60 (m, 2H, OCH_2_CH_2_CH_2_), 1.78 (m, 4H, OCH_2_CH_2_), 3.70 (s, 2H, CH_2_C=N), 3.92 (t, J = 6.33, 2H, CH_2_O), 3.97 (t, J = 6.03, 2H, CH_2_O), 6.81 (d, J = 8.45, 2H, H_ar_), 6.90 (d, J = 8.59, 2H, H_ar_), 7.11 (d, J = 8.45, 2H, H_ar_), 7.38 (m, 7H, H_ar_); ^13^C RMN (50 MHz, CDCl_3_) δ 22.56 (CH_2al_), 28.80 (CH_2al_), 28.84 (CH_2al_), 30.53 (CH_2_C=N), 67.70 (CH_2_O), 67.76 (CH_2_O), 114.62 (CH_ar_), 114.87 (CH_ar_), 124.45 (Cq_ar_), 126.50 (CH_ar_), 127.95 (CH_ar_), 128.55 (CH_ar_), 129.68 (CH_ar_), 133.55 (Cq_ar_), 140.63 (Cq_ar_), 158.39 (O-Cq_ar_), 158.56 (O-Cq_ar_), 158.64 (C=N), 160.73 (C=O). Anal. (C_26_H_26_N_2_O_4_∙1/4PhOH∙1/4EtOH) C, N, H.

**4,5-Dihydro-3-(4-(5-(phenoxy)pent-1-yloxy)benzyl)-1,2,4-4H-oxadiazol-5-one** (**C7**) was obtained according to the general procedure from **3f** (0.11 g, 0.34 mmol) in 45% yield as a brown oil after 4h of reflux and purification (EtOAc/CH_2_Cl_2_, 2:98, v/v): IR (neat, cm^-1^) 3132 (NH), 1784 (C=O), 1600 (C=C_ar_); ^1^H RMN (200 MHz, CDCl_3/_CD_3_OD) δ 1.53 (m, 2H, OCH_2_CH_2_CH_2_), 1.77 (m, 4H, OCH_2_CH_2_), 3.69 (s, 2H, CH_2_C=N), 3.90 (m, 4H, CH_2_O), 6.83 (m, 5H, H_ar_), 7.17 (m, 4H, H_ar_); ^13^C RMN (50 MHz, CDCl_3_) δ 22.67 (CH_2al_), 28.91 (CH_2al_), 28.96 (CH_2al_), 30.61 (CH_2_C=N), 67.54 (CH_2_O), 67.78 (CH_2_O), 114.43 (CH_ar_), 115.10 (CH_ar_), 120.53 (CH_ar_), 123.89 (Cq_ar_), 129.38 (CH_ar_), 129.92 (CH_ar_), 158.60 (O-Cq_ar_), 158.82 (O-Cq_ar_), 158.93 (C=O), 161.18 (C=N). Anal. (C_22_H_23_N_3_O_4_∙1/6PhOH∙1/3EtOH) C, N, H.

**4,5-Dihydro-3-(4-(5-(phenanthren-9-yloxy)pent-1-yloxy)benzyl)-1,2,4-4H-oxadiazol-5-one** (**C8**) was obtained according to the general procedure from **3g** (0.10 g, 0.23 mmol) in 48% yield as a red oil after 6h of reflux and purification (EtOAc/CH_2_Cl_2_, 2:98, v/v): IR (neat, cm^-1^) 3400 (NH), 1763 (C=O), 1623 (C=C_ar_); ^1^H RMN (200 MHz, CDCl_3_) δ 1.80 (m, 6H, OCH_2_(CH_2_)_3_), 3.68 (s, 2H, CH_2_C=N), 3.91 (t, J = 5.86, 2H, CH_2_O), 4.17 (t, J = 6.15, 2H, CH_2_O), 6.81 (m, 3H, H_ar_), 7.07 (m, 2H, H_ar_), 7.55 (m, 5H, H_ar_), 8.30 (m, 1H, H_ar_), 8.53 (m, 2H, H_ar_), 9.96 (br s, 1H, NH); ^13^C RMN (50 MHz, CDCl_3_) δ 22.92 (CH_2al_), 28.94 (CH_2al_), 29.71 (CH_2al_), 30.69 (CH_2_C=N), 67.68 (CH_2_O), 67.82 (CH_2_O), 102.56 (CH_ar_), 114.08 (Cq_ar_), 115.21 (CH_ar_), 122.50 (CH_ar_), 123.06 (Cq_ar_), 126.30 (CH_ar_), 126.43 (CH_ar_), 126.65 (Cq_ar_), 126.85 (CH_ar_), 127.08 (CH_ar_), 127.32 (CH_ar_), 127.40 (CH_ar_), 129.93 (CH_ar_), 131.24 (Cq_ar_), 132.95 (Cq_ar_), 152.78 (O-Cq_ar_), 158.44 (O-Cq_ar_), 158.90 (C=O), 160.99 (C=N). Anal. (C_28_H_26_N_2_O_4_∙1.5H_2_O) C, N, H.

**REFERENCES**

1. Cha SS, Lee D, Adams J, Kurdyla JT, Jones CS, et al. (1996) High-resolution X-ray crystallography reveals precise binding interactions between human nonpancreatic secreted phospholipase A2 and a highly potent inhibitor (FPL67047XX). J Med Chem 39: 3878-3881.

2. Frisch MJ, Trucks GW, Schlegel HB, Scuseria GE, Robb MA, et al. (2001) Gaussian 98 (Revision A.1x). Gaussian, Inc, Pittsburgh PA,: 2747-2757.

3. Plocki S, Aoun D, Ahamada-Himidi A, Tavarès-Camarinha F, Dong CZ, et al. (2005) Molecular modeling, design, and synthesis of less lipophilic derivatives of 3-(4-tetradecyloxybenzyl)-4H-1,2,4-oxadiazol-5-one (PMS1062) specific for group II enzyme. Eur J Org Chem 2005: 2747-2757.

4. Honig B, Nicholls A (1995) Classical electrostatics in biology and chemistry. Science 268: 1144-1149.
